# Assessing extensive coronary artery disease using a myocardial jeopardy score based on coronary CT: long-term prognostic value

**DOI:** 10.1007/s10554-025-03520-9

**Published:** 2025-10-17

**Authors:** Andreas A. Giannopoulos, Alexia Rossi, Dimitrios V. Moysidis, Jan A. Schaab, Stjepan Jurisic, Tobia Albertini, Alessandro Candreva, Barbara E. Stähli, Dominik C. Benz, Ronny R. Buechel, Philipp A. Kaufmann, Aju P. Pazhenkottil

**Affiliations:** 1 Department of Nuclear Medicine, Cardiac Imaging, University Hospital Zurich, Raemistrasse 100, Zurich, CH-8091 Switzerland; 2 Department of Cardiology, University Heart Center, University Hospital Zurich, Zurich, CH-8091 Switzerland

**Keywords:** Coronary artery disease, Coronary computed tomography angiography, British cardiovascular intervention society jeopardy score, Prognostic significance, Clinical outcomes, Major adverse cardiovascular events

## Abstract

**Abstract:**

The British Cardiovascular Intervention Society Jeopardy Score (BCIS-JS) is an established, simplified scoring system for assessing coronary artery disease (CAD) extensiveness with excellent agreement between the invasive and the computed tomography (CT) angiography-based scores. Originally developed for use during invasive coronary angiography, it supports procedural planning and risk stratification by estimating the extent of myocardium at risk. The computed tomography (CT)-based version (CT-BCIS-JS) has shown excellent agreement with the invasive score. We aimed to investigate the value of the CT-BCIS-JS in predicting long-term outcomes. This retrospective single-center study included 337 patients referred for coronary CT angiography (CCTA). CT-BCIS-JS was calculated using a purpose-developed online calculator, and patients were divided into extensive and non-extensive CAD (BCIS ≥ 6 and BCIS < 6, respectively). The primary outcome was the composite of all-cause death and nonfatal myocardial infarction (MI) and the secondary outcome was major adverse cardiovascular events (MACEs), including all-cause mortality, MI, unstable angina requiring hospitalization, and coronary revascularization. Kaplan-Meier curves and multivariable-Cox regression analyses were utilized to compare outcomes between groups. Of the 337 patients, 249 (73.9%) patients had BCIS-JS < 6 and 88 (26.1%) patients had BCIS-JS ≥ 6. Overall, the primary outcome occurred in 47 (13.9%) patients over a median follow-up of 6.8 years (interquartile range 5.7–8.5 years). Thirthy-eight of them (43.2%) had BCIS-JS ≥ 6 compared to 9 (3.6%) in the BCIS-JS < 6 group (adjusted hazard ratio [aHR]: 11.15; 95% CI 4.97–24.99; *p* < 0.001). The secondary outcome occurred in 77 patients (87.5%) who had BCIS-JS ≥ 6 compared to 20 (8.0%) in the BCIS-JS < 6 group (aHR: 19.92; 95% CI: 11.40-34.81; *p* < 0.001). CT-BCIS-JS allows excellent risk stratification in patients with known or suspected CAD. Anatomically extensive CAD identified with the CT-BCIS-JS is a strong predictor of higher mortality, nonfatal MI, and MACE.

**Graphical abstract:**

**Figure:**
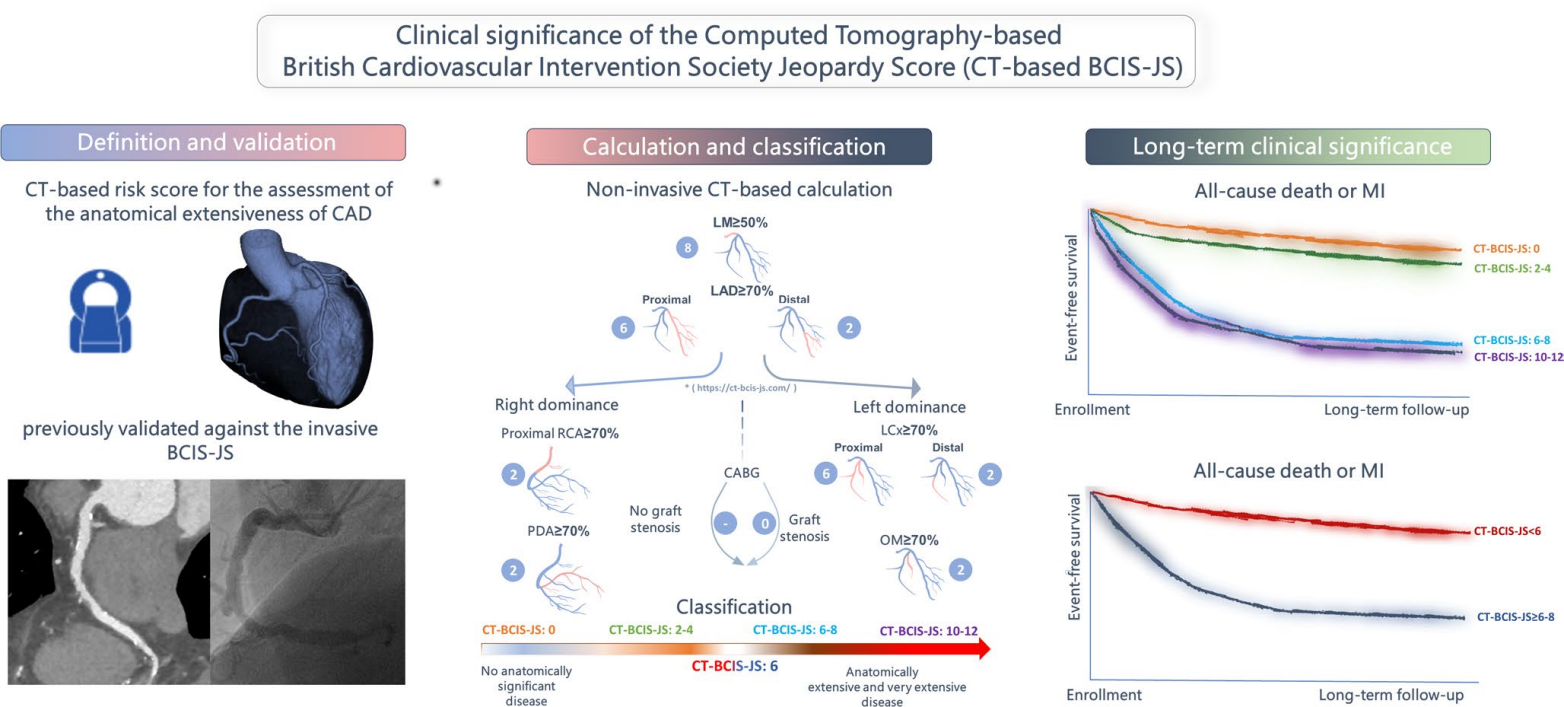
Graphical representation of the definition, calculation and classification as well as the long-term prognostic significance of the coronary CT Angiography-Based British Cardiovascular Intervention Society Jeopardy Score (CT-BCIS-JS). The figure illustrates the method used to quantify myocardial jeopardy, the scoring algorithm, and its application in risk stratification for extensive coronary artery disease (CAD). Kaplan-Meier survival curves demonstrate the long-term clinical impact of the CT-BCIS-JS, with higher scores associated with poorer event-free survival

**Supplementary Information:**

The online version contains supplementary material available at 10.1007/s10554-025-03520-9.

## Introduction

Non-invasive cardiac imaging with coronary computed tomography angiography (CCTA) is increasingly recommended as the diagnostic modality for patients with a low to moderate likelihood of obstructive stable coronary artery disease (CAD), according to current European and American clinical practice guidelines [1, 2]. While invasive coronary angiography has traditionally been the gold standard for assessing coronary stenoses, CCTA offers comparable prognostic utility and diagnostic accuracy, even in patients with complex or multi-vessel disease [3, 4]. Non-invasive, reliable, and standardized identification of patients with anatomically extensive (high-risk) CAD - associated with more frequent major adverse events and worse outcomes - may be of significant clinical importance [5–7].

To this end, several semi-quantitative angiographic risk scores have been developed, demonstrating prognostic superiority over mere binary evaluations of the presence of obstructive stenosis [8–11]. The invasive British Cardiovascular Intervention Society Jeopardy Score (BCIS-JS) is an established angiographic scoring system that quantifies the extent of myocardium at risk [12, 13]. Grading lesions with ≥ 70% diameter stenosis on a scale from zero (non-significant CAD) to twelve (very extensive CAD), it enables rapid and accurate identification of extensive CAD [14]. More recently, the CCTA-based BCIS-JS (CT-BCIS-JS) was developed, showing excellent correlation and almost perfect agreement with the invasive jeopardy score [15]. This emerging tool offers a simple and precise method for personalized assessment of CAD extensiveness.

The invasive BCIS-JS has been assessed for its predictive value in patients with CAD following percutaneous coronary intervention (PCI), showing positive outcomes in this context [14]. However, the utility of the non-invasive counterpart - CT-BCIS-JS to predict long-term prognosis and adverse cardiovascular events is still unexplored. This study aims to determine the clinical utility of the CT-BCIS-JS by examining its prognostic predictive value.

## Patients and methods

### Study population

This retrospective, single-center study included patients from the hybrid CCTA/single-photon emission computed tomography-myocardial perfusion imaging registry of the Department of Nuclear Medicine at the University Hospital of Zürich [16, 17]. The protocol as well as the inclusion and exclusion criteria have been previously reported in a study which examined the impact of cardiac hybrid imaging on long-term clinical outcome [18]. The present research work focused solely on the anatomical imaging test of the same population (CCTA) and used follow-up data, which have been partially previously reported [18]. Patients with known or suspected CAD referred for CCTA between 2005 and December 2008 and available follow-up data were included. Reasons for patient referral were investigation of CAD in asymptomatic patients or patients with typical or atypical symptoms. The study protocol was approved by the institutional review board (the local Ethics Committee of the Canton of Zurich) and the need for additional written informed consent was waived. The study was performed in accordance with the general principles outlined in the Declaration of Helsinki and the rules of good clinical practice (GCP).

### CCTA scans and calculation of CT-BCIS-JS

Patients were scanned on a 64-slice CT scanner (LightSpeed VCT; GE Healthcare) with either helical or prospective ECG triggering, as previously reported in detail [19, 20]. CCTA images were evaluated by two experienced, European Association of Cardiovascular Imaging (EACVI) qualified cardiologists using dedicated software (CardIQXpress-Auto Coronary Analysis, GE Healthcare). Prior to calculation of the CT-BCIS-JS, two readers (A.A.G. and A.P.P) each with > 10 years of experience in cardiovascular imaging assessed the image quality of the CCTA data on a dedicated workstation (CardIQ Xpress-Auto Coronary Analysis, GE Healthcare). A five-point Likert scale to determine the quality of the examination as follows: 1: non-diagnostic (severe artifacts), 2: poor (pronounced artifacts or low contrast), 3: fair (moderate artifacts), 4: good (mild artifacts), 5: excellent (absence of artifacts). CCTA scans with a Likert scale score of 1 and 2 were excluded for further analysis.

The CT-BCIS-JS was calculated for all patients on-site using a purpose-developed online calculator (https://www.ct-bcis-js.com/). The calculation process has been previously described in detail and a case example is presented in **Video 1** (calculation algorithm also shown in **Figure ****S1**) [15]. Briefly, this semi-quantitative score evaluates the extent of myocardium at risk based on significant coronary stenoses (≥ 70%, or ≥ 50% in the left main) across 18 coronary segments, as defined by the Society of Cardiovascular Computed Tomography (SCCT). The score ranges from 0 to 12 and incorporates adjustments for coronary dominance, anatomical variations, and bypass grafts. The higher the score, the more extensive the CAD. A CT-BCIS-JS of 6 or higher indicates extensive CAD [21]. The average time required to calculate this score is less than 2 min per patient [15].

### Definition of outcomes and follow-up

Follow-up data were obtained from telephone calls to patients and their physicians, if possible. In addition, medical charts were retrospectively reviewed for clinical cardiac events. The primary outcome was the occurrence of hard clinical events defined as the composite of all-cause mortality and nonfatal myocardial infarction (MI). The combination of all-cause death and non-fatal MI in the primary outcome was selected to include hard clinical endpoints with distinct and complementary prognostic value. Fatal MI was not separately included, as such events are already encompassed within all-cause mortality. Secondary outcome included major adverse cardiovascular events (MACEs), including all-cause mortality, MI, unstable angina requiring hospitalization, and coronary revascularization (either percutaneous coronary intervention [PCI] or CABG). All patients who underwent revascularization within the first 30 days after the non-invasive test were excluded because the treatment decision might have been caused by the test result itself. This exclusion criterion was set to minimize bias from test-triggered management decisions, ensuring that outcome events reflected disease progression rather than early intervention based on the CT findings. All-cause mortality was defined as death from any cause and MI was defined according to the Second and Third universal definition of myocardial infarction (depending on the time of the follow-up) [18, 22, 23].

### Statistical analysis

Baseline patient characteristics were examined by presence of myocardial ischemia and extent-severity of CAD. Patient characteristics were compared using the Chi-squared test or Fisher’s exact test for categorical variables and the 2-sided Student’s t-test or the one-way ANOVA for continuous variables. When assumptions of normality were not met, the non-parametric Mann-Whitney U test or the Kruskal–Wallis test were used. Categorical variables are presented by frequencies and percentages (%), and continuous variables are summarized as mean ± standard deviation (SD) or median (1st −3rd quartile).

A time-to-event analysis was performed to assess whether anatomically extensive CAD was associated with worse clinical outcome. For analyses of the extent and presence of high-risk CAD in relation to outcomes, the CT-BCIS-JS was used. Patients were firstly categorized in two groups according to the absence or presence of extensive CAD (BCIS-JS < 6 and ≥ 6, respectively). Additional analyses evaluated also the association between outcomes and CT-BCIS-JS subcategories (CT-BCIS-JS = 0; CT-BCIS-JS = 2–4; CT-BCIS-JS = 6–8; CT-BCIS-JS = 10–12).

Patients were censored at the time of the event or last contact with the study investigator. Event rates were presented using the Kaplan-Meier curves and compared by the Log-rank test. Analyses were adjusted for a priori specified set of baseline covariates, including age, sex, body mass index (BMI), known history of CAD, hypertension, diabetes mellitus, hypercholesterolemia, family history of CAD, and current smoking. For each testing strategy, adjusted hazard ratios (HRs) and 95% confidence intervals (CIs) were computed using Cox regression models. The proportional hazards assumption required by the Cox model was investigated using the Schoenfeld residuals. A p value of < 0.05 was considered to indicate statistical significance. Statistical analysis was performed with IBM SPSS Statistics 25.0 (IBM Corp., New York, NY, USA) and STATA 17.0 (StataCorp, Texas, USA).

## Results

### Patient population

A total of 414 patients with completed follow-up were considered for inclusion in the present analysis. Accounting for early revascularization, 39 patients were excluded. A total of 38 patients with a Likert scale score of 1 − 2 were also excluded. Hence, 337 patients with a mean age of 61.7 ± 11 years were finally included and 216 of them (64%) were males. Overall, 249 (73.9%) patients had CT-BCIS-JS < 6 (189 [56.1% of total population] of them had CT-BCIS-JS = 0) and 88 (26.1%) patients had CT-BCIS-JS ≥ 6 (60 [17.8% of total population] of them had CT-BCIS-JS = 10–12). Baseline demographic characteristics based on CT-BCIS-JS are presented in Table 1. More comprehensive baseline characteristics for each CT-BCIS-JS subcategory are summarized in Table S1 of the supplementary appendix. Patients with BCIS ≥ 6 were slightly older and had more frequently history of known CAD and coronary revascularization with respectively more frequent use of anti-ischemic medications.

### Main outcomes

**Fig. 1:**
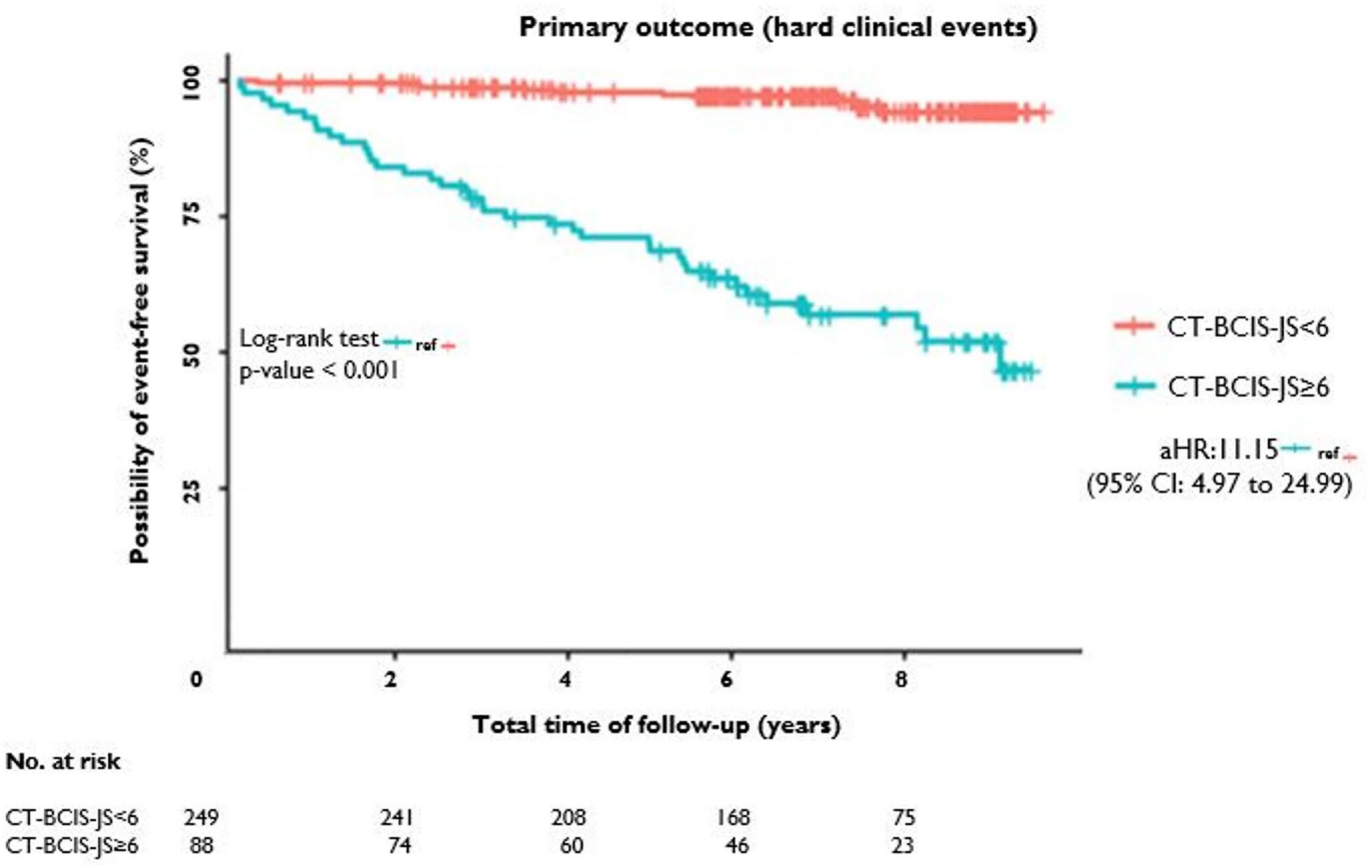
Kaplan-Meier survival estimates for hard clinical event-free survival in patients stratified by high (≥ 6; red line) vs. low (< 6; blue line) CT-BCIS-JS. The primary outcome was hard clinical events defined as a composite of all-cause mortality and nonfatal myocardial infarction. CT-BCIS-JS denotes coronary CT Angiography-Based British Cardiovascular Intervention Society Jeopardy Score

**Fig. 2:**
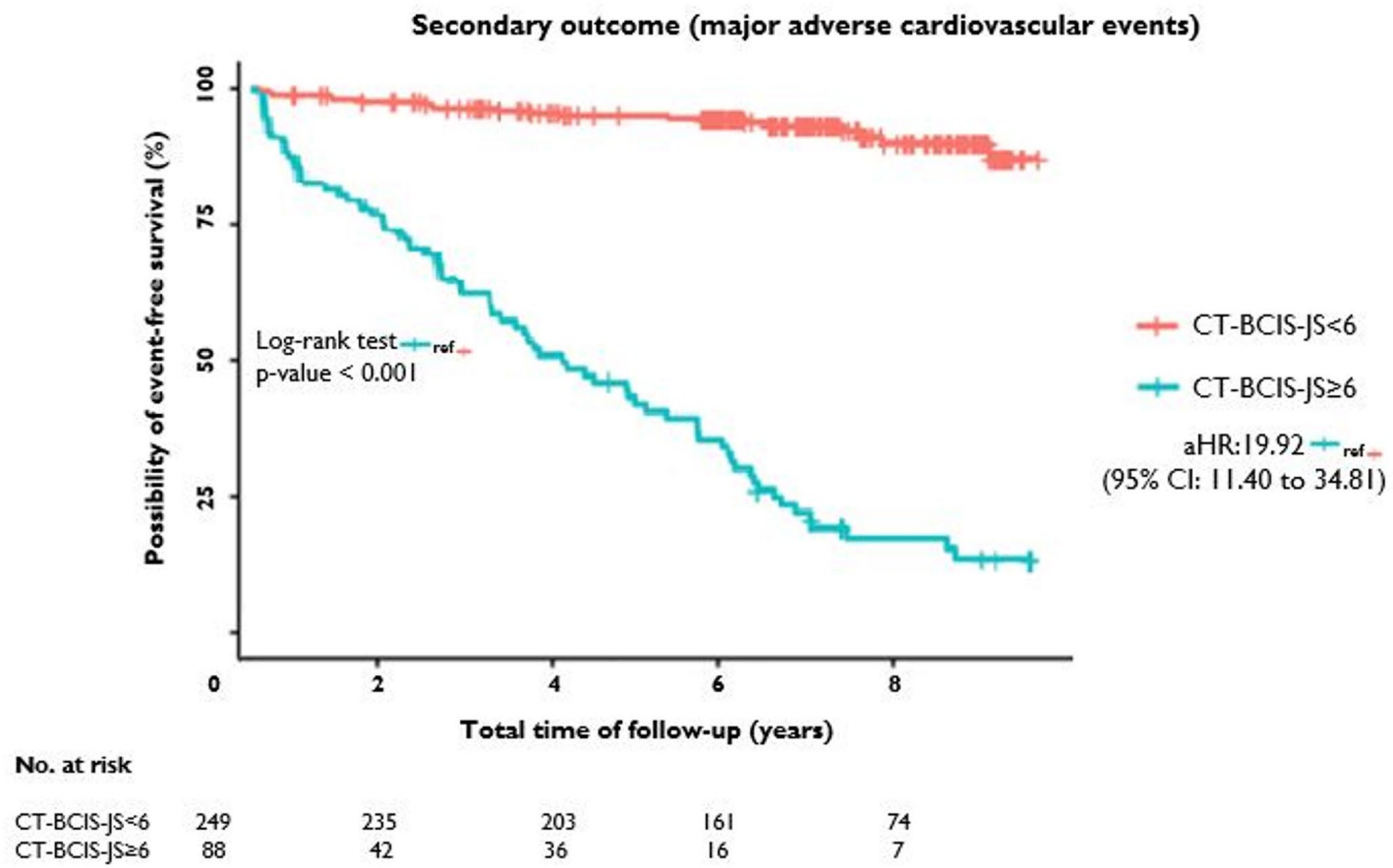
Kaplan-Meier curves illustrating all-cause mortality, myocardial infarction, unstable angina requiring hospitalization, and coronary revascularization (secondary outcome) in patients with extensive CAD (CT-BCIS-JS ≥ 6; red line) versus those with lower jeopardy scores (< 6; blue line). Patients with extensive CAD had a significantly higher risk the secondary outcome (log-rank *p* < 0.001). CT-BCIS-JS denotes coronary CT Angiography-Based British Cardiovascular Intervention Society Jeopardy Score

Over a median follow-up of 6.8 years (interquartile range [IQR] 5.7 to 8.5 years), 47 (13.9%) patients suffered a hard clinical event, defined as death from any cause and nonfatal MI. Of the 47 patients, 38 (43.2% within group) had CT-BCIS-JS ≥ 6 compared to 9 (3.6%) in the CT-BCIS-JS < 6 group (unadjusted hazard ratio [HR]: 14.05; 95% confidence interval [CI] 6.79 to 29.08; *p* < 0.001). After adjustment for pre-specified baseline variables, the HR for the primary outcome was 11.15 (95% CI: 4.97 to 24.99). Regarding the composite secondary outcome, MACE was found in 97 (28.8%) patients. Of them, 77 (87.5% within group) had CT-BCIS-JS ≥ 6 in comparison to 20 (8.0%) in the CT-BCIS-JS < 6 group (unadjusted HR: 21.50; 95% CI: 13.03 to 35.46; *p* < 0.001). The adjusted HR [aHR] was similar to the unadjusted HR: 19.92; 95% CI: 11.40 to 34.81; *p* < 0.001). Time-to-event analysis for the primary and the secondary outcome are depicted in Figs. 1 and 2.

**Table 1:** Baseline characteristics of the patients based on CT-BCIS jeopardy score

Parameters	CT-BCIS-JS < 6 (*N* = 249; 73.9%)	CT-BCIS-JS ≥ 6 (*N* = 88; 26.1%)	*P*-value
*Demographics*			
Age (Years) - Means(± SD)	60.2 (10.7)	65.7 (10.9)	**< 0.001**
Female Sex – No (%)	100 (40.2)	21 (23.9)	**0.006**
BMI(Kg/m^2^) - Means(± SD)	26.5 (4.7)	27.8 (4.3)	0.351
*Reason for referral*			
Atypical symptoms/No complaints	225 (90.4)	80 (90.9)	0.139
Typical angina	17 (6.8)	7 (8.0)	0.724
Only dyspnea	7 (2.8)	1 (1.1)	0.375
*Underlying Diseases – No (%)*			
CAD	27 (10.8)	34 (38.6)	**< 0.001**
Previous myocardial infarction	7 (2.8)	10 (11.4)	**0.003**
Previous CABG	6 (2.4)	9 (10.2)	**0.002**
Previous PCI	1.6 (6.4)	1(47.5)	**< 0.001**
Obesity	32 (20.9)	9 (16.1)	0.435
Diabetes mellitus	31 (12.4)	16 (18.2)	0.182
Dyslipidemia	102 (41)	58 (65.9)	**< 0.001**
Hypertension	138 (55.4)	56 (63.6)	0.180
Positive family history of CAD	80 (32.1)	32 (36.4)	0.468
Current smoking	62 (24.9)	35 (39.8)	**0.008**
*Sum of risk factors - Means(± SD)*	1.6 (1.1)	2.2 (1.3)	**< 0.001**
*Medications – No (%)*	***N = 96 (69.1%)***	***N = 43 (30.9%)***	
Aspirin	51 (53.6)	31 (72.1)	**0.036**
Statin	29 (30.2)	21 (48.8)	**0.034**
ACEs	31 (32.3)	25 (58.1)	**0.004**
Beta-blocker	33 (34.4)	25 (58.1)	**0.009**
Nitrates	4 (4.2)	2 (4.7)	0.897

*ACEs, angiotensin-converting enzyme inhibitors; BCIS-JS, British Cardiovascular Intervention Society Jeopardy Score; CABG, coronary artery bypass grafting; CAD, coronary artery disease; PCI, percutaneous coronary intervention; BMI body mass index

*ǂ Data on medications were available only for 139 patients. The numbers in parentheses represent percentages relative to this number.*

### Subgroup analyses

**Fig. 3:**
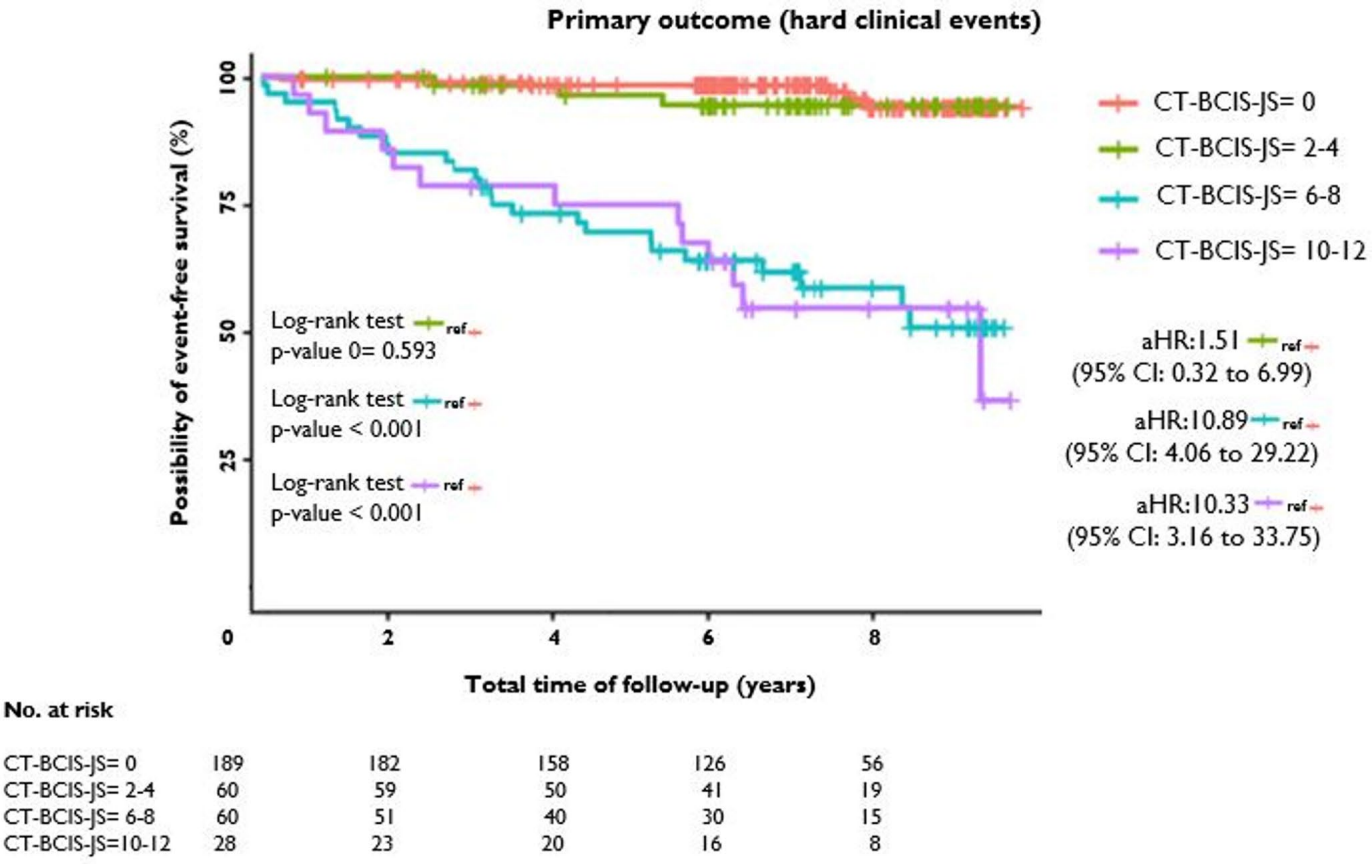
Kaplan-Meier estimates for hard clinical event-free survival, stratified by CT-BCIS-JS subcategories. The higher the CT-BCIS-JS (10–12; purple line, vs. 6–8; blue line vs. 2–4; green line vs. 0; red line) the higher the incidence of the primary outcome defined as a composite of all-cause mortality and nonfatal myocardial infarction. CT-BCIS-JS denotes coronary CT Angiography-Based British Cardiovascular Intervention Society Jeopardy Score

When patients were further divided according to CT-BCIS-JS in 4 pre-specified categories, those with CT-BCIS-JS = 6–8 and CT-BCIS-JS = 10–12 demonstrated a statistically significant difference compared to BCIS-JS = 0 for both the primary (Fig. 3) and the secondary outcome respectively. Table S2 summarizes the unadjusted and adjusted hazard ratios for all outcomes.

## Discussion

In this retrospective single-center study, we demonstrated that patients with extensive CAD, identified using the CT-BCIS-JS, have an increased risk of mortality and rates of MACE as compared to patients with non-extensive CAD. To our knowledge, this is the first study to assess the prognostic value of the CT-based BCIS-JS, emphasizing its utility as a clinical tool not only for the assessment of the extent of jeopardized myocardium pertinent to obstructive CAD, but also for risk stratification.

This study approached extensive CAD as an important clinical indicator that should be easily and reliably recognized by non-invasive imaging. The clinical significance of extensive CAD, identified using invasive coronary angiography but also employing CCTA, has been previously described in several studies [5, 9, 24]. In a retrospective analysis of the Prospective Multicenter Imaging Study for Evaluation of Chest Pain (PROMISE) trial, high-risk CAD, defined as left main stenosis ≥ 50% stenosis or ≥ 70% stenosis of 3-vessels or 2-vessel CAD involving the proximal left anterior descending artery, was associated with more frequent invasive interventions and adverse events [5]. In a more recent sub-analysis of the International Study of Comparative Health Effectiveness with Medical and Invasive Approaches (ISCHEMIA) trial, although patients with left main disease were excluded, the authors also showed a graded association between CAD severity and adverse cardiovascular events [24]. Our results build on and extend these findings by demonstrating, in an observational setting, the prognostic value of a global metric that quantifies the extent of jeopardized myocardium.

The invasive BCIS-JS, long proposed as a simplified angiographic scoring system for identifying patients with extensive CAD, has limited data supporting its prognostic significance [17]. Its ability to predict mortality was retrospectively demonstrated using the ‘Revascularization Index’, which compares pre- and post-PCI BCIS-JS scores [14]. The completeness of revascularization using the jeopardy score was an independent predictor of all-cause mortality, and the pre-PCI BCIS-JS score was directly related to mortality [14]. The respective CT-based score has been recently validated, showing excellent correlation and agreement with its invasive counterpart, along with very high reproducibility [15]. Several other CT-based CAD scoring systems, such as the CT-based modified Duke Prognostic Index, the Segment Stenosis Score (SSS) and the Segment Involvement Score (SIS), have proven effective in distinguishing complex CAD and predicting outcomes. However, each system has its own limitations, and few have been validated against their invasive reference standard [8, 10]. The BCIS-JS provides a distinct approach to risk stratification compared to established CT-based scores. While SIS and SSS reflect the overall burden and severity of coronary atherosclerosis by counting involved segments or weighing stenosis severity, they do not incorporate information on the amount of myocardium at risk. The extensively studied SYNTAX scores I and II are comprehensive angiographic grading tools that incorporate anatomical and clinical factors to assess CAD complexity [11, 25]. Although the invasive SYNTAX scores have prognostic implications, the CT-based SYNTAX scores are primarily used in revascularization decision-making, but their calculation is complex and time-consuming [13, 26]. In contrast, the CT-derived BCIS-JS estimates the extent of jeopardized myocardium based on the presence of high-grade stenoses in major coronary territories, thereby linking anatomical disease with potential functional impact. This may enhance its clinical relevance, particularly in identifying patients more likely to benefit from revascularization. In our study, CT-based BCIS-JS was significantly associated with long-term cardiovascular events, supporting its value as a practical, functionally oriented risk stratification tool following abnormal CCTA. While we did not directly compare BCIS-JS with other CT-based scoring systems, its conceptual focus on myocardial jeopardy offers complementary prognostic insight beyond anatomical burden alone.

In contrast to Coronary Artery Disease Reporting and Data System (CAD-RADS), which primarily focuses on the most severe stenosis and serves as a standardized framework for reporting and management guidance, the CT-BCIS-JS provides a comprehensive, semi-quantitative estimate of the total myocardium at risk. While CAD-RADS grades the lesion and includes modifiers for high-risk plaque features, stents, or grafts, it does not account for lesion distribution, coronary dominance, or graft patency, nor does it offer a summative burden of disease. The CT-BCIS-JS systematically evaluates all major coronary territories, including native vessels and bypass grafts, integrating anatomical complexity and extent of ischemic myocardium into a reproducible numeric score. The CT-BCIS-JS can complement and extend the information provided by CAD-RADS, offering a more complete assessment of coronary disease burden and aiding in revascularization planning. It is further important to note that the CT-BCIS-JS does not quantify total plaque burden, but rather estimates the extent of myocardium at risk based on the location and severity of obstructive lesions. In clinical practice, both total plaque burden and CT-BCIS-JS may be used in a complementary fashion, as they provide distinct yet synergistic information relevant for risk stratification, ischemia assessment, and revascularization planning.

The primary advantage of the CT-BCIS-JS lies in its ability to quickly and easily provide reliable estimates of CAD extensiveness using an online calculator. This system is particularly valuable for assessing patients with left main lesions and bypass grafts, which are often omitted in other angiographic scores. A CT-BCIS-JS score of ≥ 6, corresponding to approximately 50% of the left ventricular myocardium at risk, was used as an inclusion criterion in the REVIVED-BCIS2 trial [21]. Although the trial did not formally validate this threshold, its use highlights the clinical relevance of the ≥ 6 cutoff for identifying patients with substantial myocardial jeopardy. Our findings support the prognostic value of this threshold; patients with CT-BCIS-JS scores ≥ 6 experienced significantly more events and worse outcomes, while those with scores of 0 and 2–4 had similarly low event rates. This non-linear pattern likely reflects the categorical structure of the score, which was designed to distinguish between non-extensive and extensive CAD rather than to provide a continuous gradient of risk. The limited number of score categories and small sample sizes in the intermediate groups may also have contributed to the observed clustering of outcomes. By focusing primarily on stenoses in the proximal coronary tree, devoid of complicated plaque analyses and incorporating coronary dominance, the CT-based BCIS-JS was demonstrated to hold substantial prognostic value. This makes it a potential effective tool for identifying patients with extensive CAD, improving personalised risk stratification, and informing need for subsequent testing as well as management decisions.

This study should be interpreted in the context of certain acknowledged limitations. First, despite the application of multivariable adjustments, our results remain susceptible to unrecognized biases inherent in retrospective observational studies and unmeasured confounding factors. Additionally, our study was single-centered and had a relatively limited sample size. However, the significant number of events allowed for a robust assessment of the predictive value of the CT-BCIS-JS. In addition, our cohort included patients with both known and previously undiagnosed CAD, which may have introduced heterogeneity in terms of baseline risk, clinical management, and secondary prevention strategies. Although statistical models were adjusted for history of CAD and major cardiovascular risk factors, residual confounding related to differences in care pathways cannot be entirely excluded. Moreover, this study included many patients with extensive CAD, constituting a group likely not representative of the currently lower-risk patients undergoing CCTA. Furthermore, MIs were defined based on the previous definitions, which presents some differences compared to the fourth universal definition that is currently used. Additionally, coronary calcium scoring, and detailed plaque quantification were not available, two important parameters also associated with adverse outcomes. While our study was not designed to compare CT-BCIS-JS directly against CAD-RADS or other existing scoring systems, our results underscore its independent prognostic value. Future studies are warranted to evaluate its incremental benefit over established frameworks in larger and more diverse populations.

## Conclusion

Anatomically extensive CAD identified using the CT-BCIS-JS, a non-invasive, simplified angiographic scoring system, was associated with increased rates of mortality, nonfatal MI, and MACE. Further studies involving larger and more contemporary patient populations with suspected obstructive CAD are needed to evaluate the potential role of CT-BCIS-JS in guiding therapeutic decision-making for extensive CAD.

## Supplementary Information

Below is the link to the electronic supplementary material.

Supplementary Material 1

Supplementary Material 2

Supplementary material 3: Video 1. Case example of the CT-BCIS-JS computation. Using the online calculator (left panel) each predefined coronary segment is sequentially scored as obstructive or not by assessing in parallel the axial and multiplanar reconstructions of the coronary CT angiography (CCTA; right panel). The left main (LM) coronary artery presents no lesions → the proximal left anterior descending (pLAD) presents a lesion with ≤ 70% luminal stenosis → the distal left anterior descending (dLAD) presents no lesions → the dominant diagonal branch (DG) presents a lesion with ≤ 70% luminal stenosis → the right coronary artery (RCA) supplies the posterior descending artery, hence this is a coronary tree with right dominance → the proximal right coronary artery (pRCA) presents a lesion with > 70% luminal stenosis→ the obtuse marginal (OM; in right dominant systems represented by the left circumflex artery) presents lesions with ≤ 70% luminal stenosis → no presence of coronary artery bypass grafts (CABG): The total CT-BCIS-JS is 4.

## Data Availability

No datasets were generated or analysed during the current study.
