# Supplementary material for: Assessing extensive coronary artery disease using a myocardial jeopardy score based on coronary CT: long-term prognostic value
Source: Int J Cardiovasc Imaging. 2025 Oct 17;41(11):2173–82. doi: 10.1007/s10554-025-03520-9 (PMC12628459; doi:10.1007/s10554-025-03520-9)
Supplement: Supplementary file 1 — Supplementary Material 1 [file 10554_2025_3520_MOESM1_ESM.docx]

**Supplemental Figures**

## Supplemental Figure 1. CT-British Cardiovascular Intervention Society Jeopardy Score Calculator

| Row | Coronary | Instructions | Score |
| --- | --- | --- | --- |
| 1 | LMCA | If ≥50%, score 8 and go to row 11  If <50%, score 0 and go to row 2 |  |
| 2 | LAD before DG | If ≥70%, score 6 and go to row 5  If <70%, score 0 and go to row 3 |  |
| 3 | LAD after DG | If ≥70%, score 2 and go to row 4  If <70%, score 0 and go to row 4 |  |
| 4 | Major DG | If ≥70%, score 2 and go to row 5  If <70%, score 0 and go to row 5 |  |
| 5 |  | If LCX dominant, go to row 8  If RCA dominant, go to row 6 |  |
| 6 | RCA before PDA | If ≥70%, score 4 and go to row 10  If <70%, score 0 and go to row 7 |  |
| 7 | PDA | If ≥70%, score 2 and go to row 10  If <70%, score 0 and go to row 10 |  |
| 8 | LCX before OM | If ≥70%, score 6 and go to row 14  If <70%, score 0 and go to row 9 |  |
| 9 | LCX after OM | If ≥70%, score 2 and go to row 10  If <70%, score 0 and go to row 10 |  |
| 10 | Major OM | If ≥70%, score 2 and go to row 14  If <70%, score 0 and go to row 14 |  |
| 11 |  | If LCX dominant, score 4 and go to row 14  If RCA dominant, score 0 and go to row 12 |  |
| 12 | RCA before PDA | If ≥70%, score 4 and go to row 14  If <70%, score 0 and go to row 13 |  |
| 13 | PDA | If ≥70%, score 2 and go to row 14  If <70%, score 0 and go to row 14 |  |
| 14 |  | Previous CABG? If yes, go to row 15  If no, go to row 21 |  |
| 15 | LAD-Graft beyond DG | If <70% graft lesion, score -4 and go to row 16  If ≥70%, poor run-off or n/a, score 0, go to row 16 |  |
| 16 | DG-Graft | If <70% graft lesion, score -2 and go to row 17  If ≥70%, poor run-off or n/a, score 0, go to row 17 |  |
| 17 | OM-Graft | If <70% graft lesion, score -2 and go to row 18  If ≥70%, poor run-off or n/a, score 0, go to row 18 |  |
| 18 | Dominant LCX-Graft beyond OM | If <70% graft lesion, score -4 and go to row 19  If ≥70%, poor run-off or n/a, score 0, go to row 19 |  |
| 19 | RCA-Graft before PDA | If <70% graft lesion, score -4 and go to row 21  If ≥70%, poor run-off or n/a, score 0, go to row 20 |  |
| 20 | PDA-Graft | If <70% graft lesion, score -2 and go to row 21  If ≥70%, poor run-off or n/a, score 0, go to row 21 |  |
| **21** | **TOTAL SCORE** | Add filled-in scores and enter (range 0-12) |  |

LMCA: left main coronary artery; LAD: left anterior descending coronary artery; DG: diagonal branch; RCA: right coronary artery; PDA: posterior descending artery; Cx: circumflex artery, OM: obtuse marginal.

*adapted by *Perera D, Stables R, Booth J, Thomas M, Redwood S. The balloon pump-assisted coronary intervention study (BCIS-1): rationale and design. Am Heart J 2009;158:910e916.e2.*
