# Supplementary material for: Assessing extensive coronary artery disease using a myocardial jeopardy score based on coronary CT: long-term prognostic value
Source: Int J Cardiovasc Imaging. 2025 Oct 17;41(11):2173–82. doi: 10.1007/s10554-025-03520-9 (PMC12628459; doi:10.1007/s10554-025-03520-9)
Supplement: Supplementary file 2 — Supplementary Material 2 [file 10554_2025_3520_MOESM2_ESM.docx]

**SUPPLEMENTARY MATERIAL**

**Supplementary Tables**

**Table S1.** Baseline characteristics of the patients based on subcategorization of the CT-BCIS-JS

| Parameters | CT-BCIS-JS: 0  (N=189; 56.1 %) | CT-BCIS-JS: 2-4 (N=60; 17.8%) | CT-BCIS-JS: 6-8 (N=60; 17.8%) | CT-BCIS-JS: 10-12 (N=28; 8.3%) | P-value | Significant differences |  |
| --- | --- | --- | --- | --- | --- | --- | --- |
| Demographics |  |  |  |  |  |  |  |
| Age (Years) - Means(±SD) | 59.0 (±11.1) | 64.0 (±8.3) | 65.1 (±11.7) | 66.9 (±9.10) | **<0.001** | **0 vs 2-4; 0 vs 6-8; 0 vs 10-12** |  |
| Female Sex – No (%) | 86 (67.9) | 14 (21.5) | 14 (21.5) | 7 (10.1) | **<0.001** | **0 vs 2-4; 0 vs 6-8; 0 vs 10-12** |  |
| BMI(kg/m^2^) - Means(±SD) | 26.6 (±4.6) | 26.0 (±5.1) | 27.6 (±4.8) | 28.4 (±3.2) | 0.067 | - |  |
| Reasons for referral |  |  |  |  |  |  |  |
| Atypical symptoms / No complaints | 168 (88.9) | 58 (96.7) | 54 (90.0) | 25 (89.3) | 0.349 | **-** |  |
| Typical angina | 16 (8.5) | 1 (1.7) | 4 (6.7) | 3 (10.7) | 0.286 | **-** |  |
| Only dyspnea | 5 (2.6) | 1 (1.7) | 1 (1.7) | 1 (3.6) | 0.921 | **-** |  |
| Underlying Diseases – No (%) |  |  |  |  |  |  |  |
| Coronary artery disease (CAD) | 10 (5.3) | 18 (30.0) | 23 (38.3) | 10 (35.7) | **<0.001** | **0 vs 2-4; 0 vs 6-8; 0 vs 10-12** |  |
| Previous myocardial infarction | 2 (1.1) | 5 (8.3) | 8 (13.3) | 2 (1.4) | **<0.001** | **0 vs 2-4; 0 vs 6-8; 2-4 vs 10-12; 6-8 vs 10-12** |  |
| Previous CABG | 2 (1.1) | 4 (6.7) | 8 (13.3) | 1 (3.6) | **<0.001** | **0 vs 2-4; 0 vs 6-8; 2-4 vs 6-8** |  |
| Previous PCI | 7 (3.7) | 10 (16.7) | 13 (21.7) | 5 (17.9) | **<0.001** | **0 vs 2-4; 0 vs 6-8; 0 vs 10-12** |  |
| Obesity | 26 (22.4) | 6 (16.2) | 7 (18.4) | 2 (11.1) | 0.635 | - |  |
| Diabetes mellitus | 18 (9.5) | 12 (20) | 12 (20) | 5 (17.9) | 0.069 | - |  |
| Dyslipidemia | 79 (41.8) | 23 (38.3) | 37 (61.7) | 21 (75.0) | **<0.001** | **0 vs 6-8; 0 vs 10-12; 2-4 vs 6-8; 2-4 vs 10-12** |  |
| Hypertension | 107 (56.5) | 30 (50.0) | 35 (58.3) | 22 (78.6) | 0.088 | - |  |
| Positive family history of CAD | 62 (3.6) | 18 (30.0) | 21 (35.0) | 11 (39.3) | 0.839 | - |  |
| Current smoking | 48 (25.4) | 14 (23.3) | 23 (38.3) | 12 (42.9) | 0.063 | - |  |
| Sum of risk factors - Means(±SD) | 1.6 (1.0) | 1.6 (1.1) | 2.1 (1.3) | 2.5 (1.2) | **<0.001** | **0 vs 6-8; 0 vs 10-12; 2-4 vs 10-12** |  |
|  |  |  |  |  |  |  |  |
| Medication – No (%) ^ǂ^ | **73 (52.5%)** | **23 (16.5%)** | **28 (20.1%)** | **15 (10.7%)** |  |  |  |
| Aspirin | 37 (50.7) | 14 (60.9) | 20 (71.4) | 11 (73.3) | 0.159 | - |  |
| Statin | 17 (23.3) | 12 (52.2) | 14 (50.0) | 7 (46.7) | **0.013** | **0 vs 2-4;** **0 vs 6-8; 0 vs 10-12** |  |
| ACEs | 20 (27.4) | 11 (47.8) | 18 (64.3) | 7 (46.7) | **0.006** | **0 vs 6-8** |  |
| B-Blocker | 22 (30.1) | 11 (47.8) | 15 (53.6) | 10 (66.7) | **0.020** | **0 vs 10-12** |  |
| Nitrates | 3 (4.1) | 1 (4.3) | 1 (3.6) | 1 (6.7) | 0.970 | - |  |
| ** ACEs, angiotensin converting enzymes; BCIS-JS, British Cardiovascular Intervention Society Jeopardy Score; CABG, coronary artery bypass grafting; PCI, percutaneous coronary intervention; CAD, coronary artery disease; BMI, body mass index*  *ǂ*  *Data on medications were available only for 139 patients. The numbers in parentheses represent percentages relative to this number.* | | | | | | |  |

**Table S2.** Unadjusted and adjusted hazard ratios of all outcomes

|  | Outcomes | Primary (Hard Events) | | | |  | Secondary (MACE) | | | |
| --- | --- | --- | --- | --- | --- | --- | --- | --- | --- | --- |
| Predictive parameters |  | **Unadjusted HRs (95% CIs)** | **P-value** | **Adjusted HRs (95%CIs)** | **P-value** |  | **Unadjusted HRs (95% CIs)** | **P-value** | **Adjusted HRs (95%CIs)** | **P-value** |
| CT-BCIS-JS |  |  |  |  |  |  |  |  |  |  |
| BCIS-JS≥6 _(ref: BCIS-JS<6)_ |  | 14.05 (6.79 - 29.08) | **<0.001** | 11.15 (4.97 - 24.99) | **<0.001** |  | 21.50 (13.03 - 35.46) | **<0.001** | 19.92 (11.40 - 34.81) | **<0.001** |
| BCIS-JS=2-4 _(ref: BCIS-JS=0)_ |  | 1.59 (0.39 – 6.37) | 0.073 | 1.51 (0.32 – 6.99) | 0.073 |  | 2.30 (0.92 – 5.72) | 0.073 | 2.21 (0.79 – 6.19) | 0.130 |
| BCIS-JS=6-8 _(ref: BCIS-JS=0)_ |  | 15.47 (6.34 – 37.75) | **<0.001** | 10.89 (4.06 – 29.22 | **<0.001** |  | 25.42 (13.15 – 49.12) | **<0.001** | 25.36 (11.79 – 54.51) | **<0.001** |
| BCIS-JS=10-12 _(ref: BCIS-JS=0)_ |  | 16.48 (6.26– 43.40) | **<0.001** | 10.33 (3.16 – 33.75) | **<0.001** |  | 62.88 (26.47 – 149.34) | **<0.001** | 41.33 (15.39 – 111.65) | **<0.001** |
